# Prevalence and factors associated with second hand smoke exposure among a sample of pregnant women in Cairo, Egypt

**DOI:** 10.1186/s12905-023-02821-2

**Published:** 2024-02-26

**Authors:** Elizabeth R. Stevens, Erin L. Mead-Morse, Kareem Labib, Linda G. Kahn, Sugy Choi, Scott E. Sherman, Cheryl Oncken, Natasha J Williams, Tom Loney, Omar El Shahawy

**Affiliations:** 1 Department of Population Health, New York University Grossman School of Medicine, New York, NY USA; 2 Department of Medicine, UConn Health School of Medicine, Connecticut, USA; 3 Department of Obstetrics and Gynecology, Ain Shams University School of Medicine, Cairo, Egypt; 4 Department of Pediatrics, New York University Grossman School of Medicine, New York, USA; 5 Public Health Research Center, New York University in Abu Dhabi, Abu Dhabi, United Arab Emirates; 6 College of Medicine, Mohammed Bin Rashid University of Medicine and Health Sciences, Dubai Health, Dubai, United Arab Emirates; 7 School of Global Public Health, New York University, New York, USA

**Keywords:** Egypt, Second hand smoke exposure, Hookah, Smoking, Pregnancy, Smoking water pipes

## Abstract

**Purpose:**

This study estimated the prevalence of and factors associated with secondhand smoke (SHS) exposure, and assessed attitudes and knowledge about SHS among pregnant women in Cairo, Egypt.

**Methods:**

Pregnant women in the third trimester were recruited to participate in a survey assessing tobacco smoking and SHS exposure during their current pregnancy. Participants were recruited from three antenatal clinics in Cairo, Egypt, from June 2015 to May 2016. We examined differences in sociodemographic characteristics and SHS exposure, attitudes, and knowledge by smoking/SHS status. We used multivariable ordinary least squares regression to examine the association between husbands’ smoking and pregnant women’s mean daily hours of SHS exposure, adjusting for women’s smoking status, age group, education, and urban (vs. suburban/rural) residence.

**Results:**

Of two hundred pregnant women aged 16–37 years, about two-thirds (69%) had a husband who smoked tobacco. During their current pregnancy, most women reported being non-smokers (71%), and 38% of non-smokers reported being SHS-exposed. Non-smokers exposed to SHS tended to live in more rural areas and have husbands who smoked in the home. In adjusted analyses, having a husband who smoked was significantly associated with a greater mean number of hours of SHS exposure per day exposed, and this difference was driven by husbands who smoked in the home (p < 0.001*)*. Women in the SHS-exposed group were less likely than other groups to agree that SHS exposure was harmful to their own or their future child’s health; however, all groups agreed that SHS was harmful to newborn health.

**Conclusion:**

Among our sample of pregnant women in Cairo, Egypt, there was a high rate of SHS exposure as well as misconceptions about the safety of SHS exposure to a developing fetus. Our findings suggest a need for targeted education and gender-sensitive messaging about SHS exposure, along with improved enforcement of existing tobacco control policies.

**Supplementary Information:**

The online version contains supplementary material available at 10.1186/s12905-023-02821-2.

## Plain Language Summary

Exposure to secondhand smoke (SHS) remains a major contributor to health problems in pregnant women and their children. Using a survey, this study sought to estimate how many pregnant women in Cairo Metropolitan Area, Egypt, were exposed to SHS and the factors contributing to that exposure, and to assess attitudes towards SHS. During their current pregnancy, 38% of non-smokers reported being exposed to SHS. Non-smokers exposed to SHS tended to live in more rural areas and have husbands who smoked in the home. Having a husband who smoked as well as a husband who smoked in the home was significantly associated with a greater average number of SHS exposure hours per day. Women in the SHS-exposed group were less likely than other groups to agree that SHS exposure was harmful to their own or their future child’s health; however, all groups agreed that SHS was harmful to newborn health. Among pregnant women in Cairo, Egypt, there is a high rate of SHS exposure—often driven by SHS exposure in the home—as well as misconceptions about the safety of SHS exposure to a developing fetus. There is a need for targeted education and gender-sensitive messaging about SHS exposure along, with improved enforcement of existing tobacco control policies.

**Supplementary Information:**

The online version contains supplementary material available at 10.1186/s12905-023-02821-2.

## Introduction

Exposure to tobacco smoke remains a major contributor to adverse health outcomes in pregnant women and their children. Secondhand smoke (SHS) exposure among women who are of childbearing age in Egypt is reportedly very high, with estimates of around 60% at the home and more than 50% at work [1–4]. Maternal smoking, as well as exposure to SHS, is associated with increased risks for adverse birth outcomes and maternal complications [5]. SHS exposure carries similar risks for pregnant women compared with active smoking, [5–7] and women exposed to SHS are more likely to give birth to infants with a lower birth weight, [8, 9] reduced birth length, [10] smaller head circumference, [11] and stillbirth [12]. There is no safe level of exposure to SHS for non-smokers.

While smoking among women in Egypt is relatively rare (0.5%), many men smoke (36.0%) and current rates of smoking in Egypt remain high with 41.3% of households having at least one smoker, contributing to about 24 million non-smokers being exposed to SHS [13]. Cigarettes are the main form of combustible tobacco used in Egypt, followed by hookah (i.e., waterpipe instrument used for smoking tobacco), which is locally referred to as shisha [14]. In rural areas, upwards of 15% of men smoke hookah [15]. SHS exposure is more intense from smoking hookah compared with cigarettes, and the exposure to toxicants from a typical hookah tobacco smoke session can be up to 200 times that of cigarettes [16–20].

To combat SHS exposure, Egypt has implemented smoke-free policies as well as complex taxation policies [21]. Despite these rules, there is little policy enforcement. The lack of policy enforcement comes at the expense of women and children, who are usually victims of high levels of SHS exposure in Middle Eastern countries [3, 9, 22–26]. Moreover, many of these policies aim only to limit smoking in public places; however, women are still vulnerable due to the high prevalence and duration of SHS exposure in their homes [3, 26].

Knowledge regarding the impact of SHS exposure in pregnancy is lower among people with low socioeconomic status (SES) and in developing countries, especially Arab countries that exhibit higher smoking rates. Egyptian culture encompasses social norms similar to those of other Arab countries, with conservative attitudes and male-dominated family structures. Distinct gender roles in conservative cultures allow men to preside over the social practices and norms at home [27]. Due to the presence of gender inequality at the community and interpersonal levels, it is difficult for women to negotiate to establish smoke-free homes [28, 29]. Attitudes toward SHS exposure and knowledge about its harms is an initial step to facilitate a change in SHS avoidance behaviors, [30] but this has been poorly studied in Egypt.

The literature from other Middle Eastern countries indicates the potential for high exposure to SHS in these countries; [3–26, 31] however, the full extent of exposure to SHS and to what extent women are impacted by SHS due to the smoking behavior of their husband in the home is not reflected in current national surveys, as the majority of national surveys do not evaluate the risks of tobacco intake methods such as hookah, which is extremely popular in Egypt. This is important in order to guide tobacco control efforts in the country. Furthermore, there are no published studies that assess SHS exposure due to both cigarettes and hookah among pregnant women in Egypt. To fill these gaps, this study sought to estimate the prevalence of and factors associated with SHS exposure, and assess attitudes toward SHS among pregnant women in Cairo Metropolitan Area.

## Methods

### Setting and population

Pregnant women in their third trimester were recruited from antenatal clinics in one of the largest public maternity hospitals and from two private obstetric clinics in Cairo, Egypt, between June 2015 and May 2016, to participate in an interviewer-administered Arabic language survey that assessed tobacco exposure and attitudes and knowledge about SHS. The clinics serve the Cairo Metropolitan Area, which is composed of three districts that include both urban and rural communities. We approached 229 pregnant women, and those who agreed to participate were screened for eligibility. Women with chronic medical conditions before and during pregnancy such as hypertension, diabetes mellitus, heart disease, and renal disease, as well as women who were believed to be mentally or physically incapable of participating in the interview, as judged by the interviewer, were excluded from the sample. Full survey details have been reported elsewhere [32]. Informed consent was obtained from all participants, who provided signed consent prior to survey administration, and appropriate permissions were obtained from participating clinics. This research was performed in accordance with the Declaration of Helsinki and was approved by the Institutional Review Board of Ain Shams University.

### Measures

The questions used in the study survey were adapted from an instrument designed by Bloch et al. (2008), [7] which has been used across multiple countries to assess SHS exposure and tobacco use among pregnant women in low and middle income countries. This adaptation process was led by CO and other authors (OS, EM, KL) in consultation with international and local experts in tobacco use surveys (see supplement for English version of adapted survey tool). All participants answered questions about their sociodemographic characteristics, expected date of delivery, knowledge and attitudes about tobacco use, and their own SHS exposure and tobacco use, as well as tobacco use by their husbands and other household members. We collected age (years), educational level [preparatory school completed or less (< 10 years), some secondary school or greater (≥ 10 years)], employment status (employed or not employed), and residential area (urban, suburban, or rural).

#### Smoking and SHS exposure

Participants were asked about their smoking behavior and SHS exposure during their current pregnancy. Participants who indicated smoking cigarettes or hookah (i.e., shisha/waterpipe) at least monthly were considered to be smokers. Hookah smokers were asked how many bowls of tobacco they smoked using a hookah in the past month, and cigarette smokers were asked the number of days they smoked cigarettes in the past month and, on days they smoked, how many cigarettes they smoked on average. Smokers were also asked how long they had used a hookah/smoked cigarettes at this frequency (less than 6 months, 6 months to < 1 year, 1 to < 2 years, 2 years to < 3 years, 3 years to < 4 years, 4 years or longer).

To assess overall indoor SHS exposure, participants were asked, “How often are you indoors and around people who are smoking cigarettes or other types of tobacco products?” (rarely/never, sometimes, frequently, always). Participants were considered to be SHS exposed if they responded sometimes, frequently, or always, and non-SHS exposed if they responded rarely/never. Based on these two variables, we categorized participants’ smoking and SHS exposure as follows: non-smokers/non-SHS exposed, non-smokers/SHS exposed, and smokers. Participants were also asked about SHS exposure in the household: “Did any of your household members, including your husband, smoke around you in the past 30 days inside the house?” (no, yes, don’t know/not sure).

Participants were asked how many hours in a day and days in a week they are usually exposed to SHS anywhere. To create a measure of mean daily hours of overall SHS exposure, these two items were multiplied and then divided by 7. We also asked participants how many hours ago they were indoors with people smoking around them (either hookah or cigarettes). Exhaled carbon monoxide (CO) was collected at the time of the survey using Covita (piCo, now named Micro-basic) Smokerlyzer.

We collected information on husbands’ smoking behaviors with the following item: “Does your husband smoke?” (do not have a husband; no; yes, but not inside the home; and yes, smokes inside the home). We considered a husband to be a smoker if the participant responded either of the two “yes” options. We also asked about which product husbands smoke the most (cigarettes versus hookah).

#### Smoking and SHS knowledge and attitudes

To assess attitudes toward tobacco use and SHS and avoidance self-efficacy, on a 5-point Likert scale (1 = very much disagree, 2 = slightly agree, 3 = neither agree nor disagree, 4 = slightly agree, 5 = very much agree) all participants were asked how much they agreed or disagreed with the following statements: (1) “It is socially acceptable for women to smoke cigarettes”; (2) “It is socially acceptable for women to smoke shisha”; (3) “It is easy to tell people living with you not to smoke at home”; and (4) “It is easy to tell guests visiting you not to smoke at home.” Items 1 and 2 were averaged to create perceived social acceptability of smoking (Cronbach’s α = 0.99), and items 3 and 4 were averaged to create perceived ease of telling others not to smoke at home (Cronbach’s α = 0.72).

To assess knowledge of the harms of tobacco, participants were asked how much they agreed or disagreed with the following statements: (5) “A pregnant woman’s use of tobacco (shisha, cigarettes, etc.) is harmful to her or her unborn baby’s health”; (6) “A pregnant woman’s exposure to tobacco smoke of someone else is harmful to her or her unborn baby’s health”; and (7) “Tobacco smoke exposure is harmful to a newborn’s health.” Items 5–7 were kept as separate items due to poor inter-item reliability.

#### Analysis

We examined differences in sociodemographic characteristics, SHS exposure, attitudes, and knowledge by smoking/SHS status (non-smoker/non-SHS exposed, non-smoker/SHS exposed, smoker) using Kruskal-Wallis, Fisher’s exact, and Pearson’s χ^2^ tests. We also examined pregnant women’s mean daily hours of SHS exposure by husbands’ smoking status using Wilcoxon-Mann-Whitney and Kruskal-Wallis non-parametric tests.

We used multivariable ordinary least squares regression to examine the association between husbands’ smoking and pregnant women’s mean daily hours of SHS exposure, adjusting for women’s smoking status, age group, education, and urban residence (vs. suburban/rural residence). We did not adjust for employment status because: (1) a post-estimation Wald χ^2^ test showed that removing the variable from the model would not harm model fit, and (2) the variable had 11 missing values. Also, we combined suburban and rural residential locations into one category because all women in the sample living in rural areas were non-smokers. Results are reported in terms of adjusted marginal predictive means, with *p*-values from the betas. Analyses were conducted in Stata 16.1 (StataCorp, College Station, TX).

## Results

### Sociodemographic characteristics and patterns of SHS exposure

Table 1 presents participants’ sociodemographic characteristics by SHS status. A total of 200 pregnant women aged 16–37 years enrolled in the study (87% response rate). About half (47%) were 16–26 years old, and half (53%) were older than 26 years. Approximately half (52%) had at least some secondary school or higher education (≥10 years of schooling), and the majority (77%) were not employed. Nearly half of women (44%) lived in rural areas, 18% lived in suburban areas, and 38% lived in urban areas (Table 1).

**Table 1:** Participants’ sociodemographic characteristics by secondhand smoke (SHS) status (N = 200)

	Non-Smoker/ Non-SHS Exposed	Non-Smoker/ SHS Exposed	Smoker/ SHS Exposed	*p*-value
	(N = 66)	(N = 76)	(N = 58)	
*Sociodemographic Characteristics*				
Age in years,				
mean (SD) median range	25.5 (4.6) 24.0 19–35	26.4 (4.7) 25.5 16–36	29.7 (3.5) 30.0 22–37	*0.0001* ^*1*^
Age group, % (n)				
16–26 years	63.6% (42)	53.9% (41)	17.2% (10)	
> 26 years	36.4% (24)	46.1% (35)	82.8% (48)	*< 0.0001* ^*2*^
Educational attainment, % (n)				
Preparatory school or less (< 10 years)	65.2% (43)	67.1% (51)	3.5% (2)	
Secondary school or higher (≥ 10 years)	34.8% (23)	32.9% (25)	96.5% (56)	*< 0.0001* ^*3*^
Residential area, % (n)				
Urban	12.1% (8)	19.7% (15)	91.4% (53)	
Suburban	12.1% (8)	30.3% (23)	8.6% (5)	
Rural	75.8% (50)	50.0% (38)	0% (0)	*< 0.0001* ^*3*^
Employment status, % (n) (N = 189)				
Employed	17.0% (10)	26.4% (19)	24.1% (14)	
Unemployed	83.0% (49)	73.6% (53)	75.9% (44)	*0.420* ^*2*^

^1^ Tested using Kruskal-Wallis test

^2^ Tested using χ^2^ test

^3^ Tested using Fisher’s exact test

Table 2 presents participants’ SHS exposure characteristics by tobacco exposure status. Non-smokers exposed to SHS tended to live in rural areas and have a husband who smoked in the home. Smokers were older, had higher levels of education, lived primarily in urban areas, and had a husband who smoked. Of those reporting SHS smoke exposure, the median number of days exposed in the prior month was 7 with 3 h of SHS exposure on each of those days. All smokers reported being exposed to SHS. Women who smoked had the highest exhaled CO, followed by women who had SHS exposure, compared those not exposed to SHS. About two-thirds (69%) of women had a husband who smoked tobacco. Of these women, most reported that their husbands predominantly smoked cigarettes (88%) vs. hookah (12%). During their current pregnancy, most women reported being non-smokers (71%) and 38% of non-smokers were SHS exposed (Table 2).

**Table 2:** Participants’ secondhand smoke (SHS) exposure characteristics by SHS status (N = 200)

	Non-Smoker/ Non-SHS Exposed	Non-Smoker/ SHS Exposed	Smoker/ SHS Exposed	*p*-value
	(N = 66)	(N = 76)	(N = 58)	
*Secondhand Smoke Exposure*				
Husband smoking status (n = 196)				
No	81.5% (53)	8.1% (6)	3.5% (2)	
Yes	18.5% (12)	91.9% (68)	96.5% (55)	*< 0.0001* ^*3*^
Smokes, but not inside home^4^	75.0% (9)	8.8% (6)	0% (0)	
Smokes inside home^4^	25.0% (3)	91.2% (62)	100% (55)	*< 0.0001* ^*3*^
Tobacco product smoked the most by husband				
Cigarettes^4^	66.7% (8)	85.3% (58)	96.4% (53)	
Hookah^4^	33.3% (4)	14.7% (10)	3.6% (2)	*0.008* ^*3*^
Past 30-day SHS exposure inside the home				
No	75.8% (50)	2.6% (2)	1.7% (1)	
Yes	1.5% (1)	77.6% (59)	93.1% (54)	
Not sure	22.7% (15)	19.7% (15)	5.2% (3)	*< 0.0001* ^*3*^
No. hours in a day usually exposed to SHS anywhere (n = 196)				
Mean (SD) Median Range	0.2 (0.5) 0 0–2	3.0 (1.4) 3 1–7	4.2 (1.0) 4 3–7	*0.0001* ^*1*^
No. days in a week usually exposed to SHS anywhere (n = 198)				
Mean (SD) Median Range	0.6 (1.7) 0 0–7	6.2 (1.4) 7 1–7	6.8 (0.5) 7 5–7	*0.0001* ^*1*^
No. hours you were indoors with people smoking around you (n = 169)				
Mean (SD) Median Range	0.05 (0.2) 0 0–1	1.4 (1.2) 1 0–7	2.4 (0.8) 3 0–3	*0.0001* ^*1*^
Exhaled carbon monoxide (CO) level				
Mean (SD) Median Range	0.1 (0.2) 0 0–1	0.4 (0.8) 0 0–3	3.0 (1.5) 3 0–8	*0.0001* ^*1*^

^1^ Tested using Kruskal-Wallis test

^2^ Tested using χ^2^ test

^3^ Tested using Fisher’s exact test

^4^ Only among participants who reported that their husbands smoked.

In adjusted analyses (Table 3), having a husband who smoked was significantly associated with a greater mean number of hours of SHS exposure per day, and this difference was driven by husbands who smoked in the home. After adjusting for covariates, having a husband who smoked mostly hookah as compared to cigarettes was significantly associated with a greater mean number of SHS exposure hours per day exposed.

**Table 3:** Daily mean number of hours of secondhand smoke (SHS) exposure anywhere by husband’s smoking status (N = 192)

	Unadjusted^1^		Adjusted^2^	
	Mean (SD)	*p*-value	Mean (Robust SE)	*p*-value
*Model 1*: Husband’s smoking status				
Husband does not smoke	0.28 (0.78)		0.76 (0.11)	Ref
Husband smokes	3.09 (1.47)	*< 0.0001*	2.88 (0.12)	*< 0.0001*
*Model 2*: Husband’s indoor smoking status				
Husband does not smoke	0.28 (0.78)		0.68 (0.11)	Ref
Husband smokes but not inside the home	0.81 (1.07)		1.20 (0.29)	*0.085*
Husband smokes inside the home	3.34 (1.28)	*0.0001*	3.10 (0.12)	*< 0.0001*
*Model 3*: Type of product smoked by husband				
Husband does not smoke	0.28 (0.78)		0.76 (0.11)	Ref
Husband smokes mostly cigarettes	3.12 (1.47)		2.85 (0.13)	*< 0.0001*
Husband smokes mostly hookah	2.89 (1.49)	*0.0001*	3.13 (0.35)	*< 0.0001*

Note: Four women did not have spouses and were excluded. Four women were missing SHS information and were excluded

^1^ Unadjusted analysis was conducted using Wilcoxon-Mann-Whitney and Kruskal Wallis tests for non-parametric data

^2^ Adjusted analysis was conducted using linear regression with robust standard errors, adjusting for women’s current smoking status (smoker vs. non-smoker), age group (16–26 vs. 27 years or older), educational level (primary school or less vs. secondary school or higher), and urban residential area (urban vs. suburban/rural). Adjusted means were calculated using predictive margins

### Attitudes and knowledge of SHS exposure

To better understand SHS avoidance behaviors and self-efficacy, we examined knowledge and attitudes about smoking and SHS exposure (Table 4). Current smokers were significantly more likely to agree that it was socially acceptable for women to smoke (average Likert rating 4.86/5). Among non-smokers, there was low endorsement of the acceptability of women smoking overall, yet those exposed to SHS were more likely than non-exposed women to report women’s smoking as socially acceptable (1.49/5 vs. 1.04/5, respectively). All women reported that it would be difficult to tell others not to smoke in the home, but women who smoked were more likely to report it as easy. Women who did not smoke were significantly more likely to perceive SHS as harmful to a newborn’s health than smokers; however, all groups agreed that SHS was harmful to newborn health (> 4/5). Women in the non-smoker/SHS-exposed group were less likely than other groups to agree that SHS exposure was harmful to their own or their future child’s health. Overall, across all groups, women tended to believe that SHS was less harmful to a pregnant woman or developing fetus compared with smoking by the pregnant woman herself.

**Table 4:** Attitudes and knowledge about smoking of pregnant women by tobacco exposure status, mean 5-point Likert score (SD)

	Non-Smoker/ Non-SHS Exposed	Non-Smoker/ SHS Exposed	Smoker/ SHS Exposed
	(N = 66)	(N = 76)	(N = 58)
It is socially acceptable for women to smoke	1.04 (0.22)^a^	1.49 (0.96)^a^	4.86 (0.33)^a^
It is easy to tell others not to smoke at home	2.23 (0.97)^a^	2.22 (1.14)^b^	3.06 (1.20)^a,b^
A pregnant woman’s use of tobacco is harmful to her or her unborn baby’s health	4.79 (0.51)^a^	4.22 (1.07)^a^	4.12 (0.65)^a^
A pregnant woman’s exposure to tobacco smoke of someone else is harmful to her or her unborn baby’s health	4.03 (0.80)^a^	3.32 (1.01)^a,b^	3.93 (0.37)^b^
Tobacco smoke exposure is harmful to a newborn’s health	4.73 (0.62)^a^	4.45 (1.00)^b^	4.16 (0.45)^a,b^

^1^Attitudes and knowledge were measured on a 5-point Likert scale: 1 = very much disagree, 5 = very much agree

* Note: means with the same superscript letter are statistically significantly different from each other at *p* < 0.05. Tested using Kruskal-Wallis test.

## Discussion

This study is one of the first to explore SHS exposure from multiple tobacco products, including both cigarettes and hookah, among pregnant women in Egypt. Study findings show that more than half of pregnant women visiting the antenatal clinic were exposed to SHS. Consistent with the most recent national Global Adult Tobacco Survey (GATS) conducted in Egypt, [26] the most frequent environment for SHS exposure was inside the home. Overall, many women were exposed to SHS anywhere every day for multiple hours a day. High rates of exposure were particularly common among women from rural areas. While women were aware of the risks of SHS exposure on newborns in the home, they were less aware of the harms when a mother is exposed to SHS during pregnancy. Despite the small sample size of our study, our findings were very similar to the GATS national survey regarding perceptions of tobacco use harms [26].

Based on the results of our study, we suggest that misconceptions among pregnant women about the harms of SHS exposure highlights an important avenue for intervention to educate and empower women to reduce SHS exposure and improve pregnancy outcomes. Among the women surveyed, there was strong agreement that a woman smoking is harmful to her developing fetus and that SHS exposure was harmful to newborns. However, there was significantly less awareness of the potential harms of a pregnant woman’s SHS exposure to herself or her future child. Understanding where the gaps are can inform directed national campaign messaging to focus on SHS and how it impacts pregnancy health and prenatal development. Because of women’s heightened concern for their future children’s health, this approach may be more effective than a general message that smoking and SHS are harmful to health overall. Decreasing misconceptions about the harms of SHS is important given the high prevalence of smoking in Egypt, where about 70% of Egyptian households allow smoking in their homes and 80% of women report SHS exposure in their homes in the past month [26]. SHS exposure is typically even higher with the use of certain tobacco products such as waterpipes (i.e., hookah), which is prevalent in Egypt and the Middle East [18, 27, 33].

Education campaigns directed toward pregnant women alone, however, are unlikely to eliminate maternal exposure to SHS. As seen in this population, home exposure represents a significant driver of SHS exposure. Indeed, women in our study had low confidence in their ability to implement or enforce indoor smoking bans at home. As in other Middle Eastern countries that tend to have a more conservative culture and patriarchal society, women are not fully empowered in Egyptian society from a social perspective. The gender inequality within families is particularly notable. The Global Gender Gap Index (GGGI) for Egypt (0.64 points) is lower than most countries, ranking 129 out of 146 countries globally [34]. Distinct gender roles, in which men take precedence over women in shaping the social practices at home, [24] may lead to a perceived inability by women to ask family members not to smoke in the home [23]. As observed in Egypt and other Middle Eastern countries, women often feel they have limited autonomy over SHS exposure in the home [3, 9, 22–25]. Indeed, a primary obstacle to SHS avoidance has been cited as having men in the household who smoke [22, 23]. Consequently, a majority of SHS smoke exposure occurs at home [3–25, 35]. This is particularly apparent in more rural and lower SES populations that are often more culturally conservative and more traditional, as can be seen in this study and previous research in the region, [9] where women in rural and low SES areas are more likely to be exposed to SHS in the home. Successful interventions promoting SHS avoidance behaviors have included a combination of improving both attitude and self-efficacy [25, 36]. Without changes to both factors, behavior change is unlikely to occur. In this way, a woman’s knowledge about the harms of SHS does not always guarantee successful avoidance behaviors [3, 25].

Furthermore, smoke-free homes not only protect non-smokers, but lead to smoking cessation and decreased cigarette consumption [37]. In the present study, having a husband who smokes was strongly associated with SHS exposure. For this reason, gender-tailored campaigns are needed to educate men at the community level regarding the harms of SHS, to encourage smoking cessation, and to address gender inequality and provide women with a greater feeling of SHS avoidance self-efficacy. This may be particularly important in rural and lower SES populations [9, 23].

Efforts to promote women’s perceived self-efficacy through women’s empowerment need to be paired with smoking cessation campaigns that are gender-sensitive and targeted at both women and men. As seen in this study, where women who smoke were more likely to be from urban areas, increased women’s empowerment, education, and liberal norms have been associated with a higher smoking prevalence among women [38]. Therefore, as progress is made toward gender equality, specialized campaigns should be implemented to reduce the impact of empowerment on the uptake of smoking.

Pregnancy can be an important motivator for behavior change, [39, 40] and there is a need to build tobacco use counseling capacity among clinicians (i.e., physicians and nurses) delivering care to pregnant women in Egypt. Preconception and prenatal visits may be an opportunity to promote SHS education and smoking cessation among both men and women. These are windows of opportunity, according to the “teachable moment concept,” in which both men and women are more receptive to health care providers’ advice and to behavioral change interventions that can affect the health of their future child [3]. While few women report actually receiving tobacco counseling, [26] for those who do, behavioral treatments during pregnancy are consistently effective in helping pregnant women quit smoking [41, 42]. To be successful, however, health care providers need to be further trained on smoking cessation counseling and general knowledge about the importance of tobacco control. Lack of support from a health care provider has been cited as a barrier to engaging in SHS avoidance behaviors [22]. Despite this need, many providers are not equipped to provide smoking counseling or tobacco control education, with barely half of health care providers in Egypt reporting high SHS knowledge or a supportive attitude toward preventing SHS exposure [43].

In addition to increased education among patients and clinicians, there is a need for stricter enforcement of clean air laws. While enforcement appears to have been successful in some settings (e.g., metro system, airports) in Egypt, it is not consistently applied in workplaces and other public settings such as restaurants, placing even those with suitable SHS avoidance behaviors at high risk of exposure [3, 25]. While there is support for smoke-free public spaces, [44] there have been barriers to implementing non-smoking spaces, [21, 22, 43, 45] and current policies allow for non-smoking areas within public places, which has been demonstrated to be an ineffective approach to SHS protection [21, 46]. There needs to be a movement toward banning smoking entirely in public places, both indoors and outdoors, which not only leads to decreased SHS exposure, but has been shown to promote smoking cessation [47–50].

This study had a few limitations. First, we recruited pregnant women from the largest public maternity hospital and two private obstetric clinics in Cairo to minimize selection bias and maximize the generalizability of the findings. However, the small sample size and targeted recruitment approach, as well as the data collection having occurred in 2015–2016, are likely to limit the generalizability of this data. In addition, the survey did not differentiate between potential different sources of SHS outside of the home, making the assessment of factors contributing to these exposure sources infeasible. However, understanding SHS exposure sources within the home remains important, as most nonsmoking policies are limited to public places, leaving pregnant women vulnerable to SHS exposure in their households [3, 26]. The sample size is sufficiently large to identify potential patterns in SHS exposure in the home. We also excluded women with any reported complications in their current pregnancy, which might have masked additional SHS exposure in pregnancy. Second, this study is cross-sectional and retrospective in nature, capturing participants late in their pregnancy and therefore limiting our ability to assess the relations of potentially time-varying measures. Furthermore, most study measures were self-reported, which may lead to social-desirability bias, diminishing the detection of smoking exposure. Additional research examining these associations longitudinally and beginning at earlier stages of pregnancy—or even preconception—may reveal insights that could guide the targeting of anti-tobacco messages to women of reproductive age.

## Conclusion

Among pregnant women in Cairo, Egypt, there is a high rate of SHS exposure as well as misconceptions about the safety of SHS to a developing fetus. There is a need for targeted education and gender-sensitive messaging about SHS exposure along with improved enforcement of existing tobacco control policies. Counseling during clinic encounters with pregnant women and their husbands may provide a teachable moment to emphasize the importance of SHS avoidance for maternal and child health.

### Electronic supplementary material

Below is the link to the electronic supplementary material.

Supplementary Material 1

## Data Availability

The de-identified data presented in this study are available on request from the corresponding author.
