# Supplementary material for: Prevalence and factors associated with second hand smoke exposure among a sample of pregnant women in Cairo, Egypt
Source: BMC Womens Health. 2024 Feb 26;24:145. doi: 10.1186/s12905-023-02821-2 (PMC10898124; doi:10.1186/s12905-023-02821-2)
Supplement: Supplementary file 1 — Supplementary Material 1 [file 12905_2023_2821_MOESM1_ESM.docx]

**Supplementary Materials**

Questionnaire: English Version

Second Hand Smoke Exposure and Smoking in pregnancy questionnaire

1. **CO & General Information**

- Please take the CO monitor reading before starting the survey.
- DO NOT comment on the reading to the participant.

**1.0 Exhaled Carbon Monoxide Reading: ___ ppm**

- 1. What is your age? _________
  2. How many years did you go to school?
- Did not go to school
- 6 years or less (Elementary)
- 7 to 9 years (Preparatory)
- 10 to 12 years (Secondary School or Thanaweia Amma)
- 13 to 17 years (College/University)
- More than 17 years (Graduate School).
  1. What is your current occupation? ­­­­­­­­­­­________________________
- Unemployed
- denied response

1.4Where is the house that you live in located?

- Urban
- Suburban
- Rural

**II. Secondhand Smoke Exposure *(ALL pregnant women will complete this section)***

2.1 Does your spouse smoke?

- Do not have spouse **(Skip to 2.3)**
- No **(Skip to 2.3)**
- Yes, but not inside home
- Yes, smokes inside the home

2.2 What kind of tobacco does your spouse smoke the most? (Please select one)

- Cigarettes
- Shisha
- Cigar
- E-cigarettes (electronic)
- Other _______________________________
- Don’t know / Not sure

2.3 Did **any of your household members smoke, including your spouse**, around you in the past 30 days inside the house?

- No
- Yes
- Don’t know/ Not sure

2.4 How often are you indoors and around people who are smoking cigarettes or other types of tobacco products? (Choose one box below)

| **Rarely or Never** | **Sometimes** | **Frequently** | **Always** |
| --- | --- | --- | --- |

2.5 How many hours in a day are you usually exposed to secondhand smoke anywhere you are? _____hours *(note to interviewer from 1 to 16)*

2.6 How many days in a week are you usually exposed to secondhand smoke anywhere you are? ____days per week *(note to interviewer from 1 to 7*).

2.7 How many hours ago were you indoors with people who were smoking around you (either shisha or cigarettes) ?

*On a scale from **1 to 5** choose how much you agree or disagree with the following statements with 1 very much disagree and 5 very much agree

|  | **Statement** | **1**  **Very Much Disagree** | **2**  **Slightly Disagree** | **3**  **Neither Agree nor Disagree** | **4**  **Slightly Agree** | **5**  **Very**  **Much**  **Agree** |
| --- | --- | --- | --- | --- | --- | --- |
| 2.8 | It is socially acceptable for women to smoke cigarettes | □ | □ | □ | □ | □ |
| 2.9 | It is socially acceptable for women to smoke Shisha | □ | □ | □ | □ | □ |
| 2.10 | It is socially acceptable for women to smoke E-Cigarettes | □ | □ | □ | □ | □ |
| 2.11 | It is easy to tell people living with you not to smoke at home | □ | □ | □ | □ | □ |
| 2.12 | It is easy to tell guests visiting you not to smoke at home | □ | □ | □ | □ | □ |
| 2.13 | A pregnant woman’s use of tobacco (shisha, cigarettes etc) is harmful to her or her unborn baby’s health | □ | □ | □ | □ | □ |
| 2.14 | A pregnant woman exposure to tobacco smoke of someone else is harmful to her or her unborn baby’s health | **□** | □ | □ | □ | □ |
| 2.15 | Tobacco smoke exposure is harmful to a newborn’s health | □ | □ | □ | □ | □ |

**III. Smoking Behavior**

3.1 Have you ever tried or experimented with smoking any type of tobacco, even just one puff?

- No **(Go to last page- END of Survey)**
- Yes

3.2 Please circle the response that best describes you ***(pregnant woman completing the survey)***

- I use tobacco now about the same as before I found out I was pregnant.
- I use tobacco now, but more than before I found out I was pregnant.
- I use some tobacco now, but I have cut down since I got pregnant.
- I stopped using tobacco after I found out I was pregnant and I am not using any now
- I have never smoked or used tobacco *(i.e., more than 100 cigarettes, used Shisha more than 100 times)* **(Go to last page- END of Survey)**

3.3 In the past 7 days, how often did you smoke any type of tobacco?

- Everyday
- Some days
- Have not smoked in past 7 days

3.4 What kind of tobacco do you smoke? (Please select ALL that apply)

- Cigarettes **(Fill in cigarettes questions)**
- Shisha **(Fill in Shisha questions)**
- E-cigarettes (electronic) **(Fill in e-cigarettes questions)**
- Other _______________________________

3.5. What was the last tobacco product that you smoked? ---------------

3.6 How many hours ago did you smoke this product? Enter whole numbers-----

3.7 Which statement best describes your smoking of any product inside your home?

- I never smoke in the home
- I smoke in some places or at some times inside the home
- I smoke anywhere inside the home

3.8 When you knew you were pregnant, have you considered quitting smoking?

- no, never thought of it
- yes, but never actually tried
- yes, quitted but resumed smoking
- yes, and I managed to quit

3.9 Please choose how often you have used the following tobacco products **before** this current pregnancy

|  | Never | Yearly | Monthly | Weekly | Daily |
| --- | --- | --- | --- | --- | --- |
| 1. Shisha |  |  |  |  |  |
| 1. Cigarettes |  |  |  |  |  |
| 1. Electronic cigarettes |  |  |  |  |  |
| 1. Other tobacco product (list) |  |  |  |  |  |

3.10 Please choose how often you have used the following tobacco products **during** this current pregnancy *(This woman has used tobacco at any given point during the pregnancy)*

|  | Never | Yearly | Monthly | Weekly | Daily |
| --- | --- | --- | --- | --- | --- |
| 1. Shisha |  |  |  |  |  |
| 1. Cigarettes |  |  |  |  |  |
| 1. Electronic cigarettes |  |  |  |  |  |
| 1. Other tobacco product (list) |  |  |  |  |  |

3.11 How **motivated** are you to quit all tobacco use during your current pregnancy (PLEASE CIRCLE ONE RESPONSE)

| Not at all | | | | Moderately | | | | Extremely | | |
| --- | --- | --- | --- | --- | --- | --- | --- | --- | --- | --- |
| **0** | **1** | **2** | **3** | **4** | **5** | **6** | **7** | **8** | **9** | **10** |

3.12 How **confident** are you that you are able to quit all tobacco use during your current pregnancy (PLEASE CIRCLE ONE RESPONSE)

| Not at all | | | | Moderately | | | | Extremely | | |
| --- | --- | --- | --- | --- | --- | --- | --- | --- | --- | --- |
| **0** | **1** | **2** | **3** | **4** | **5** | **6** | **7** | **8** | **9** | **10** |

3.13 Is there a person whom you feel would be supportive of your efforts to quit smoking?

- Yes, specify relationship: ______________________
- NO

**IV. Shisha smoking FOR SHISHA SMOKERS ONLY**

**4.1 How many times have you smoked a hookah in your entire life?**

a. 0 times

b. 1 or more puffs, but never a full bowl or session

c. 1 time

d. 2–5 times

e. 6–15 times

f. 16–25 times

g. 26–50 times

h. 51 or more times

**4.2 How many bowls (of tobacco) did you smoke using a hookah in the past month?**

Enter Number --------------------------

**4.3 How frequently do you currently use a hookah to smoke tobacco?**

a. At least once each year, but not monthly

b. At least once each month, but not weekly

c. At least once each week, but not daily

d. At least once each day, or most days each month

e. I have never smoked a hookah

**4.4. How long have you used a hookah at this frequency?**

a. Less than 6 months

b. 6 months to less than 1 year

c. 1 to less than 2 years

d. 2 years to less than 3 years

e. 3 years to less than 4 years f. 4 years or longer

g. I have never smoked a hookah

**4.5. How old were you when you first smoked a hookah, or even tried a puff?**

a. Enter Age --------------------------------

b. I have never smoked a hookah

**4.6. Do you intend to quit using a hookah at any time?**

a. Not at all

b. In the next month

c. In the next 6 months

d. In the future

e. I do not smoke a hookah

**4.7 Please choose the most appropriate answer to the statement**

| Statement | Never | Rarely | Sometimes | Always | I have never smokes Shisha |
| --- | --- | --- | --- | --- | --- |
| I smoke hookah with friends. |  |  |  |  |  |
| I smoke hookah with Family. |  |  |  |  |  |
| I smoke hookah alone |  |  |  |  |  |
| I smoke hookah at home |  |  |  |  |  |
| I smoke hookah at cafes |  |  |  |  |  |

**V. Cigarette smoking FOR CIGARETTE SMOKERS ONLY**

5.1 In the past month, on how many days have you smoked cigarettes? -----------------

5.2 On the days you smoked, how many cigarettes did you smoke on average? -----------

**5.3. How long have you smoked cigarettes at this frequency?**

a. Less than 6 months

b. 6 months to less than 1 year

c. 1 to less than 2 years

d. 2 years to less than 3 years

e. 3 years to less than 4 years f. 4 years or longer

g. I have never smoked cigarettes

**5.4. How old were you when you first smoked a cigarette, or even tried a puff?**

a. Enter age ----------------------

b. I have never smoked cigarettes

**5.5. Do you intend to quit smoking cigarettes at any time?**

a. Not at all

b. In the next month

c. In the next 6 months

d. In the future

e. I do not smoke cigarettes

**5.6 Please choose the most appropriate answer to the statement**

| Statement | Never | Rarely | Sometimes | Always |
| --- | --- | --- | --- | --- |
| I smoke cigarettes with friends. |  |  |  |  |
| I smoke cigarettes with Family. |  |  |  |  |
| I smoke cigarettes alone |  |  |  |  |
| I smoke cigarettes at home |  |  |  |  |
| I smoke cigarettes at cafes |  |  |  |  |
| I smoke Shisha |  |  |  |  |

**VI. E-cigarette use FOR E-CIGARETTE Users ONLY**

6.1 Have you ever used electronic cigarettes?

a. Yes

b. No (If no survey is complete)

6.2 Have you used e-cigarettes during pregnancy

a. Yes

b. No (If no survey is complete)

6.3 In the past month, on how many days have you used e-cigarettes? -----------------

6.4 What brand of e-cigarettes did you use?---------------

6.5 What flavor of electronic cigarette did you use?--------------------------------

**END OF SURVEY**

PLEASE CHECK THAT YOU HAVE NOT MISSED ANY QUESTIONS

THANK THE PREGNANT WOMAN FOR PARTICIPATION AND MENTION THAT WE WILL FOLLOW UP WITH HER AFTR DELIVERY AND WISH HER A HEALTHY DELIVERY
